# The Macronova in GRB 050709 and the GRB-macronova connection

**DOI:** 10.1038/ncomms12898

**Published:** 2016-09-23

**Authors:** Zhi-Ping Jin, Kenta Hotokezaka, Xiang Li, Masaomi Tanaka, Paolo D'Avanzo, Yi-Zhong Fan, Stefano Covino, Da-Ming Wei, Tsvi Piran

**Affiliations:** 1 Key Laboratory of Dark Matter and Space Astronomy, Purple Mountain Observatory, Chinese Academy of Sciences, Nanjing 210008, China; 2 Racah Institute of Physics, The Hebrew University, Jerusalem 91904, Israel; 3 University of Chinese Academy of Sciences, Yuquan Road 19, Beijing 100049, China; 4 National Astronomical Observatory of Japan, Mitaka, Tokyo 181-8588, Japan; 5 INAF/Brera Astronomical Observatory, via Bianchi 46, I-23807 Merate (LC), Italy; 6 Collaborative Innovation Center of Modern Astronomy and Space Exploration, Nanjing University, Nanjing 210046, China

## Abstract

GRB 050709 was the first short Gamma-ray Burst (sGRB) with an identified optical counterpart. Here we report a reanalysis of the publicly available data of this event and the discovery of a Li-Paczynski macronova/kilonova that dominates the optical/infrared signal at *t*>2.5 days. Such a signal would arise from 0.05 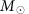 r-process material launched by a compact binary merger. The implied mass ejection supports the suggestion that compact binary mergers are significant and possibly main sites of heavy r-process nucleosynthesis. Furthermore, we have reanalysed all afterglow data from nearby short and hybrid GRBs (shGRBs). A statistical study of shGRB/macronova connection reveals that macronova may have taken place in all these GRBs, although the fraction as low as 0.18 cannot be ruled out. The identification of two of the three macronova candidates in the *I*-band implies a more promising detection prospect for ground-based surveys.

## Abstract (web summary)

A macronova is a clear signature that a short gamma-ray burst has been emitted by a compact-binary merger, but evidence of these events is so far scarce. Here, the authors report signs of a macronova in the optical afterglow of GRB050709, and find similar evidence in other three short bursts.

## Graphical abstract

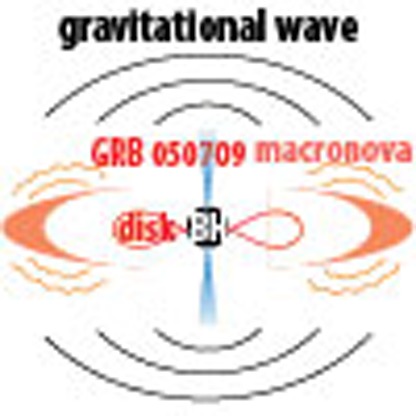

Compact object mergers are strong sources of gravitational waves (GWs) and are prime targets for the advanced LIGO/Virgo detectors^1^,^2^. It has been suggested that short Gamma-ray Bursts (sGRBs) arise from mergers in which one of the compact objects is a neutron star^3^, a scenario now favoured by a broad range of observations (for example, see refs 4, 5). In the absence of GW detection, a clear signature for the compact-binary origin of a sGRB is a Li-Paczynski macronova/kilonova: a near-infrared/optical transient powered by the radioactive decay of *r*—process material synthesized in ejecta launched during the merger^6^,^7^,^8^,^9^,^10^,^11^,^12^,^13^,^14^,^15^,^16^,^17^,^18^.

To date, the evidence of a macronova associated with sGRB 130603B is based on only a single data point^19^,^20^. The peculiar GRB 060614 was denoted as a ‘hybrid GRB (hGRB)', as its *T*_90_≈102 s groups it with long-duration GRBs, while its temporal lag and peak luminosity are within the short-duration GRB subclass^21^. Moreover, there is no evidence for an associated supernova emission^22^,^23^,^24^ down to very stringent limits. The most significant macronova evidence within this afterglow is due to a single Hubble Space Telescope (HST) observation at *t*∼13.6 days after the burst^25^. Further explorations of the afterglow allowed us to derive a tentative macronova light curve^26^. In search for further evidence for other macronovae, we explored the optical/infrared afterglows of all other nearby short and hybrid GRBs (shGRBs) in which macronova signals could have been detected. We begin with the study of GRB 050709, the first sGRB with an identified optical afterglow. Previous works have found irregularity in this afterglow and interpreted it as a jet break^27^ or as an optical flare^28^. Reanalysing the previous observations, we suggest here that this irregularity arises due to a macronova component, which in fact dominates the afterglow light curve in this burst. We then compare it with other GRBs/macronovae and explore the implications of these results to the shGRB/macronova connection.

We have identified a possible macronova in the optical afterglow data of sGRB 050709. The *I*-band light curve of this macronova candidate is remarkably similar to that of the macronova candidate of hGRB 060614 (refs 25, 26), even though the isotropic-equivalent energy (*E*_γ,iso_) of their prompt emission and the X-ray afterglow light curve are significantly different. Examination of the late-time optical–near-infrared data of all nearby short and hGRBs (*z*<0.4) for which a macronova could have been observed (six in total) revealed that there are three events GRBs 050709, 060614 and 130603B in which a macronova candidate has been detected. The three other events do not show such a signal but for each one of them there are concerns that explain this away. The appearance of a macronova candidate in three out of three (or at most six) events suggest that macronovae are ubiquitous. This supports strongly the hypothesis that compact binary mergers that are accompanied by sGRBs are the prime sites of heavy r-process nucleosynthesis. The identifications of two of those macronova candidates in the *I*-band suggest that macronova could be more easily detected in GW follow-up searches, even without a GRB trigger.

## Results

### A macronova signal associated with GRB 050709

GRB 050709 was detected by the NASA's High Energy Transient Explorer (HETE-2) and was localized by the HETE-2's Soft X-ray Camera^29^. Its prompt emission consisted of a hard spike (∼0.5 s) and an extended X-ray emission lasting ∼130 s (ref. 29). The accurate localization led to follow-up observations allowed to identify the first optical counterpart of a sGRB^27^,^30^,^31^. About 1.5 days after the trigger of sGRB 050709, Hjorth *et al.*^30^ observed it with the *Danish* 1.54 m telescope. They reported two *R*-band detections. Fox *et al.*^27^ obtained four HST exposures in the F814W-band. The HST observed the site of sGRB 050709 one year later in the same band and did not detect any signal. Covino *et al.*^31^ observed the source with the Very Large Telescope (VLT) in *V*/*R*/*I*-bands and detected the afterglow in *V*- and *R*-bands simultaneously on Jul. 12.4 UT. The optical counterpart that was localized with sub-arcsecond accuracy was in the outskirts of an irregular, late-type galaxy at a redshift of 0.16 (refs 27, 30). The host's star-formation rate, ∼0.2  per year, is much higher than that of the hosts of the two other sGRBs detected at the time, that is, 050509B and 050724, and it renders GRB 050709 to be the first sGRB occurring in a star forming ‘low-luminosity' galaxy^30^,^31^. The X-ray afterglow observations of sGRB 050709 are scarce. At *t*>200 s, there are only two significant detections by Chandra (including an X-ray flare at *t*∼16 days). Two other *Swift* (*t*∼1.6 days) and Chandra (*t*∼16.1 days) data points have a significance of ∼2*σ* (ref. 27). No radio afterglow emission has been detected^27^.

Already in 2005, Fox *et al.*^27^ noted that the early HST optical/infrared data declined as *t*^−1.25±0.09^ and then it dropped as *t*^−2.83±0.39^ between 10 and 20 days. They suggested that this arose due to a jet break. This interpretation was valid for the HST data set available at that time. Later, Watson *et al.*^28^ combined the optical/near-infrared data from the Danish 1.54 m telescope, VLT and HST, and showed that the decline is much faster: a single power law of *t*^−1.73±0.04^. A single HST data point at *t*∼9.8 days was significantly above this line and this was interpreted as a flare powered by a central engine activity. Following a reanalysis of all publicly available data, we show that the light curve is chromatic and this rules out an afterglow scenario (for example, a jet break). We find a strong evidence for the presence of a new emission component besides the regular forward shock emission, and that this component is strong not just at *t*∼9.8 days but also at earlier times. We compare the light curve to the predictions of macronova estimates and we suggest that this near-infrared excess lasting ∼10 days indicates a macronova emission.

We have analysed all publicly available optical/near-infrared data of the afterglow of sGRB 050709 (see the Methods for the details). Results of our reanalysis are generally consistent with those reported in the literature^27^,^31^. For the VLT data at ∼2.5 days, we confirm the detection in *R*- and *V*-bands. However, although previous analysis of this data yielded only an upper limit of 23.25 mag^31^, our reanalysis of the VLT *I*-band at *t*∼2.5 days resulted in a detection with a Vega magnitude of 24.1±0.2 (see the Methods for a detailed discussion of this analysis). This *I*-band observation was almost coincidental with the *R* and *V* observations with which we can reliably estimate the energy distribution of the spectrum (SED). We have also found an unpublished Gemini optical observation giving a tight 3*σ* upper limit of 25.4 mag on the *R*-band flux at *t*∼6.6 days after the burst.

**Figure 1:**
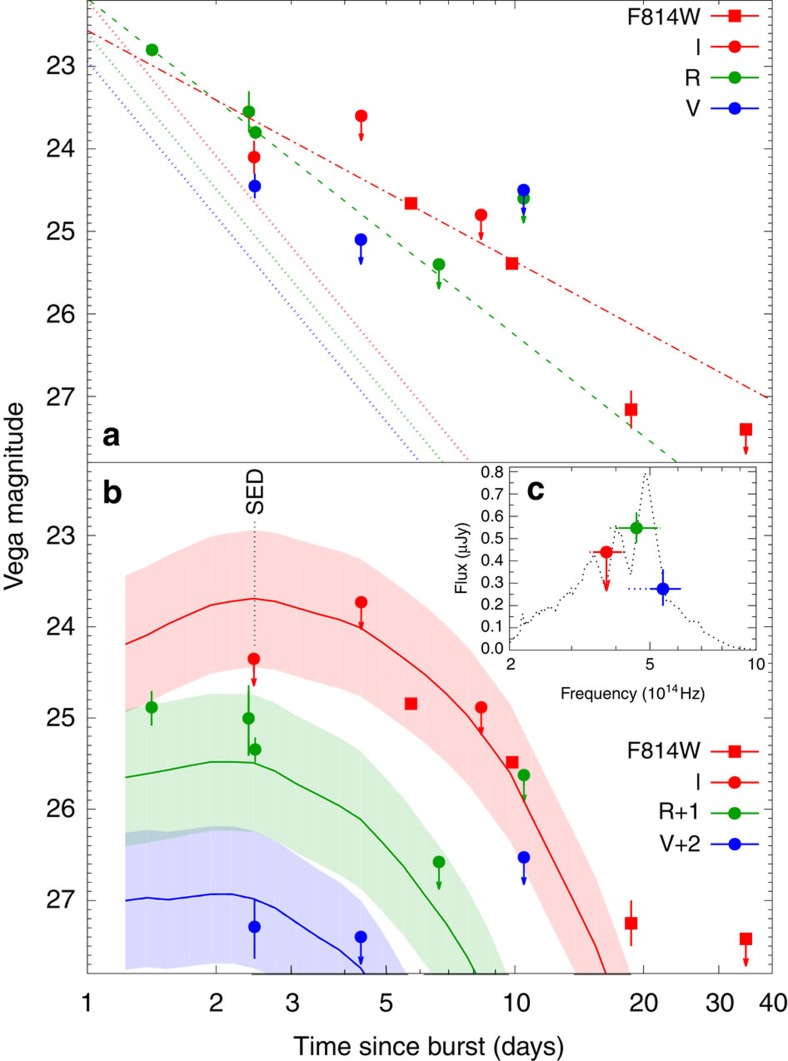
The optical observations of sGRB 050709 and the macronova component. (**a**) The fits to the *R*-band emission (green dashed line) and to the *I*-band observations from the VLT *I*-band data as well as the first two HST F814W-band data points (red dash-dotted line) yield the declines of *t*^−1.63±0.16^ and *t*^−1.12±0.09^, respectively. The dotted lines represent the ‘suggested' afterglow emission lightcurves of the GRB outflow after the jet break (that is, *t*^−2.5^ for the energy distribution index of the shock-accelerated electrons *p*∼2.5). (**b**) Shown are the residuals of the optical emission after the subtraction of a suggested fast-declining forward shock afterglow after *t*=1.4 days (dotted lines in panel (**a**)). The simulated *I*/*R*/*V*-band macronova light curves^17^ are for the ejecta from a black hole–neutron star merger, corresponding to an ejection mass of 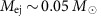 and a velocity of *V*_ej_∼0.2*c*. An uncertainty of ∼0.75 mag (the shaded region) has been adopted following Hotokezaka *et al.*^64^ (**c**) The SED of the macronova signal of sGRB 050709 measured by VLT on 12 July 2005 compared with a possible Iron line-like spectral structure adopted from Kasen *et al.*^13^ It is worth noting that all errors are 1*σ* statistical errors and the upper limits are at the 3*σ* confidence level.

The Fig. 1a depicts all the available optical/near-infrared data. The *R*-band emission decreases as *t*^−1.63±0.16^. This is consistent with the *V*-band data and with the overall fit of Watson *et al.*^28^. It is noteworthy that the new *R*-band upper limit (at *t*∼6.6 days) is consistent with the fit of Watson *et al.*^28^. On the other hand, the *I*-band emission decreases much slower, as of *t*^−1.12±0.09^, and this is consistent with the analysis of Fox *et al.*^27^ of the early HST data alone. The standard afterglow model implies an achromatic decay and hence the different behaviour in the *R*- and *I*-bands over a long timescale of ∼10 days is inconsistent with an afterglow model^32^. In fact, attempt to fit all the *I*- and *R*-band observations to a single achromatic broken power fails, with the best *χ*^2^ p.d.f. obtained of the order 10 (ignoring the HST data point at *t*∼18.7 days does not solve the problem). This strongly suggests an additional component.

**Figure 2:**
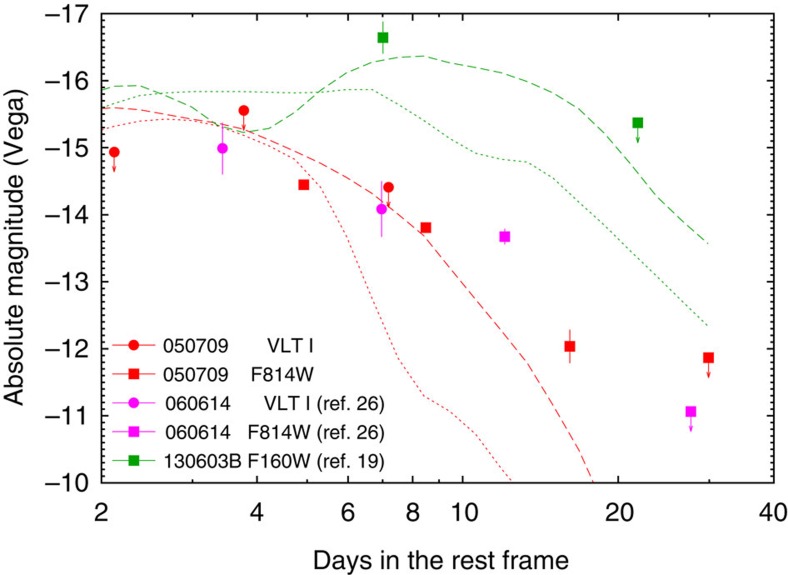
Comparison of the lightcurves of macronova candidates and theoretical models. Absolute Vega magnitudes versus rest frame time of the macronova candidates in sGRB 050709, hGRB 060614 (ref. 26) and sGRB 130603B^19^. The red dashed line is the same as the dynamical ejecta macronova model *I*-band emission presented in Fig. 1 (the green dashed line represents the *H*-band emission), whereas the red dotted line is the disk-wind ejecta macronova model *I*-band emission light curve^65^ for 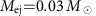 and *V*_ej_=0.07*c* (the green dotted line represents the *H*-band emission). It is noteworthy that all errors are 1*σ* statistical errors and the upper limits are at the 3*σ* confidence level.

Further information is obtained from the puzzling spectrum at *t*∼2.5 days. Here, the *R*-band flux is significantly larger than the *I*-band flux (i.e., the VLT spectrum resembles a broadened-line). This again is inconsistent with a standard afterglow model and it suggests that an additional component dominates already at this stage. Namely, any afterglow emission is subdominant already at *t*∼2.5 days. This can happen if there was an early jet break at *t*≲1.4 days, in which case the afterglow would have declines from its observed value at *t*=1.4 days as *t*^−*p*^, with *p*>2. Such a decline (with *p*=2.5, which is consistent with the X-ray spectrum) is also shown in Fig. 1a. Indeed, for *p*=2.5 and the cooling frequency *v*_c_∼2.5 × 10^16^ Hz at *t*∼2.5 days, the extrapolation of the Chandra X-ray emission into optical bands yields emission flux lower than the VLT data, consistent with the presence of a macronova emission component. Both the required fast decline rate and the jet break time are consistent with that observed in some other shGRBs and in particular in sGRB 130603B and hGRB 060614 (refs 19, 20, 33), two events displaying macronova signals. Remarkably, even without the VLT *I*-band data, Watson *et al.*^28^ already noticed that the decline is rather steep, suggesting a post jet break afterglow and that at *t*∼10 days the HST F814W-band emission was in excess of the regular forward shock afterglow emission. With the new data points, the evidence for a macronova signal is much stronger. Remarkably, this *I*/F814W macronova signal (see Fig. 2, where the suggested-afterglow component has been subtracted) is very similar to that identified in hGRB 060614 (ref. 26).

In Fig. 1b, we compared the observed lightcurves with the predictions of a macronova model. Shown are the residual of the optical emission after the subtraction of a suggested forward shock afterglow with a fast declining emission after *t*=1.4 days and the theoretical lightcurves of a macronova following a black hole–neutron star merger^17^ with 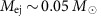 and *v*_ej_∼0.2*c*, where *c* is the speed of light, *M*_ej_ and *v*_ej_ are the ejecta mass and velocity, respectively. This is comparable but slightly smaller than the parameters used for fitting the *I*-band excess observed in the afterglow of GRB 060614 (ref. 25). Such a large amount of *r*-process material is consistent with a black hole–neutron star mergers^34^,^35^,^36^,^37^ and it also supports the hypothesis that compact object mergers are prime sites of significant production of *r*-process elements^3^,^38^,^39^,^40^,^41^,^42^,^43^,^44^. The black hole–neutron star merger scenario also has a significant implication on the prospect of establishing the GRB/GW associations in the advanced LIGO/Virgo era^45^.

The weak *I*-band emission at *t*∼2.5 days together with the almost simultaneous *R* and *V* observations, implies a puzzling broad line-like structure (see Fig. 1c for the afterglow-subtracted SED). A speculative interpretation is that this signal is due to a disk wind-driven macronova. A strong line feature can be produced by a macronova dominated by Iron^13^. Such an Iron-group-dominated macronova may arise from an accretion disk wind^46^ in which the heavier *r*-process elements are depleted, because strong neutrino irradiation from a remnant neutron star or the accretion torus can increase the electron fraction of the disk material. An interesting possibility is that the sub-relativistic neutron-rich ejecta from the compact object mergers may have a heavier or lighter composition in different directions and the resulting signal may be a combination of macronovae resulting from those (for example, see refs 47, 48). A telescope of the European Extremely Large Telescope class will be able to carry out spectroscopy of these faint signals allowing a better understanding of the phenomena.

Before concluding, we note that if we do not rely on the reanalysis of the data and adopt the afterglow interpretation of Watson *et al.*^28^, even in this case there is an *I*-band excess at 9.8 days. The most natural explanation for this excess is also a macronova and the physical parameters are similar to that adopted in the modelling of Fig. 1.

### Ubiquitousness of macronovae in afterglows of shGRBs

Following the tentative discovery of a third macronova signal, we have re-examined all nearby sGRBs and hGRBs to search for possible macronova signals. Usually the macronova optical spectrum is expected to be soft; therefore, ground-based deep *I*-band observations (ground-based *J*/*H*/*K*-band observations usually are not deep enough) as well as HST near-infrared observations are essential. The macronova candidates emerged in the sGRB 130603B, hGRB 060614 and sGRB 050709 lightcurves 1–2 weeks after the GRB triggers. At earlier times, the forward shock afterglow emission outshines the macronova component, whereas at late times the macronova emission also faded away. Hence, we need deep *I*-band or near-infrared HST observations in the time interval of ∼5–15 days. Theoretical predictions for macronovae vary significantly depending on the ejecta mass *M*_ej_, the velocity *V*_ej_, the composition, the merger types and different observing angles (for example, see Fig. 10 of ref. 14 and Fig. 9 of ref. 17 for illustration). For a reference we note that the observed signatures were ∼24.5 Vega mag at about 9 days in F160W (*H*)-band for sGRB 130603B at redshift 0.356, ∼25 Vega mag at about 13.5 days in F814W (*I*)-band for hGRB 060614 at redshift 0.125 and ∼25 mag (Vega) at about 10 days in F814W(*I*)-band for sGRB 050709 at redshift 0.16.

We focus on *Swift* and HETE-2 shGRBs at redshifts *z*≤0.4, as HST observations needed for such observations at higher redshifts are scarce^5^,^49^. The initial ‘low redshift' sample consists of sGRBs 050509B, 050709, 050724, 060502B, 061201, 071227, 080905A, 130603B, 140903A and 150101B, and hGRBs 060505 and 060614 (refs 5, 49). Unfortunately, most of these GRBs are not suitable and have to be excluded from the ‘macronova candidates' sample. There were no observations within the macronova phase for sGRBs 050724, 060502B, 071227, 080905A and 140903A. No such observations were published yet for sGRB 150101B. The *I*/near-infrared observation information of the remaining events, sGRBs 050509B, 050709, 061201 and 130603B, and hGRBs 060505 and 060614 are summarized in Supplementary Table 1 (see the Supplementary Discussion). Three events, sGRBs 050509B, 061201 and hGRB 060505 are potentially interesting but each one has its own caveat. The suggested host galaxy of sGRB 050509B is very bright and no optical counterpart had been detected. Hence, the upper limits on the ‘underlying' afterglow and macronova emission sensitively depend on the unknown location within the host galaxy (see also ref. 30). The redshift of GRB 061201 is not secure^50^ and it is possible that it was not sufficiently nearby. Using the hardness and prompt duration distribution, Bromberg *et al.*^51^ estimate that hGRB 060505 has a 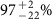 probability of being a Collapsar (see also the argument based on the location of the burst within a bright star forming region^52^ and host galaxy observations^53^).

**Figure 3:**
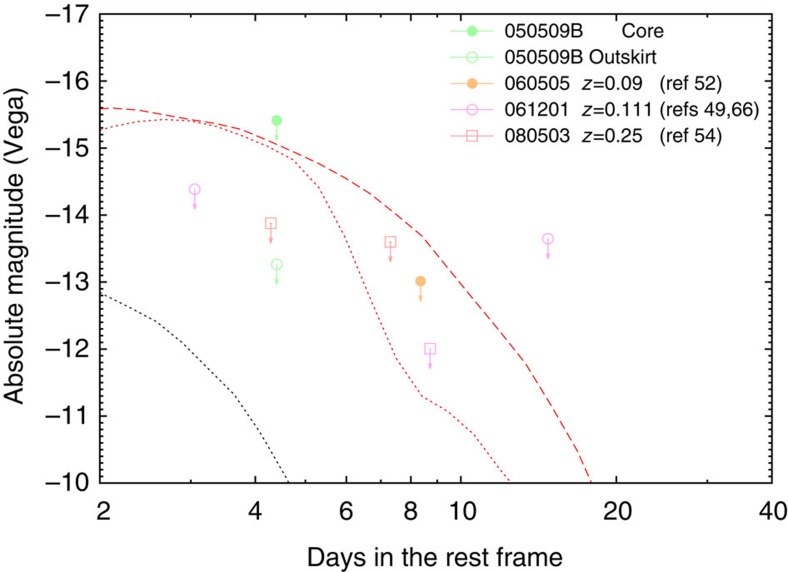
Comparison of the limits of macronova in some sGRBs and theoretical models. Absolute Vega magnitudes versus rest frame time of the I-band/F814W-band observations of sGRB 050509B, hGRB 060505 (ref. 52) and sGRB 061201 (refs 49, 66). The HST F814W 3*σ* upper limits of GRB 080503 (ref. 54) are also shown for an assumed redshift of *z*=0.25, following Kasen *et al.*^47^. It is worth noting that the Gemini *i*-band 3*σ* upper limit of sGRB 060505 was reanalysed in this work. The red dashed line is the dynamical ejecta macronova model *I*-band emission while the red solid line is the disk-wind ejecta macronova model *I*-band emission light curve, where the same model parameters in Fig. 2 are chosen. The black dotted line represents the macronova *I*-band emission expected for a double neutron star merger^14^ with 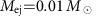 and *V*_ej_∼0.1*c*, implying that the 3*σ* upper limits reported in sGRB 050509B, hGRB 060505 (ref. 52) and sGRB 061201 are not deep enough to exclude the compact object merger origin.

Therefore, in total there are just three or at most six events that are sufficiently nearby and have sufficient data for a macronova identification. In three of those (sGRB 050709, hGRB 060614 and sGRB 130603B), there are macronovae signatures (see Fig. 2). In the three other potentially interesting events (sGRB 050509B, hGRB 060505 and sGRB 061201), there are only upper limits (see Fig. 3) but it is possible that none of them is sufficiently binding. In the most ‘optimistic' case, there are three macronovae in a sample consisting of just three events and the 95% confidence interval of the probability of a macronova taking place in a shGRB is (0.47, 1), whereas in the most ‘pessimistic' case (that is, there are three macronovae in a sample consisting of six GRBs) the 95% confidence interval for the probability is (0.18, 0.82). Therefore, the detection prospect of macronovae in merger-powered GRBs are indeed encouraging, although the fraction as low as ∼0.18 cannot be ruled out.

Within this context, it is interesting to mention GRB 080503 as well. It is not in our sample as its redshift is unknown^54^. Although no *I*-band/F814W-band or redder emission had been measured (see Fig. 3, where the upper limits on the infrared luminosity are for a redshift *z*∼0.25, as assumed by Kasen *et al.*^47^), in optical bands the afterglow was detected in the time interval of ∼1.08–5.36 days after the GRB trigger. The emission is quite blue, which is at odds with the dynamical ejecta macronova model but may be consistent with the disk-wind macronova model^47^. The potential challenge for this model is the non-identification of a nearby host galaxy as close as *z*∼0.25 in the deep HST/WFPC2 observation data of GRB 080503 (ref. 54).

It is interesting to compare now the observed features of the three macronova candidates. As far as the prompt emission is concerned, GRB 050709, a short burst with extended soft X-ray emission, bridges the gap between the canonical sGRB 130603B and the hGRB 060614 (see Table 1). The isotropic-equivalent prompt emission energy *E*_γ,iso_ of sGRB 050709 is about 30 times smaller than that of hGRB 060614 and sGRB 130603B, whereas the macronova emission of sGRB 050709 is similar to that of hGRB 060614 (see Fig. 2). The high-energy transients were powered by a relativistic jet emerging from the central engine, whereas the macronova emission arises from the *r*-process material ejected during the merger. The similarity between the macronova emission of sGRB 050709 and hGRB 060614 that had a very different energy release in the prompt phase suggests that the launch processes of the ultra-relativistic outflows and the sub-relativistic outflows are not related.

At *t*∼16 days after the trigger of sGRB 050709 there was an X-ray flare^27^,whereas at *t*>1.4 days after the trigger of hGRB 060614, the X-ray afterglow is well behaved^33^. At *t*≳1 days after the trigger of GRB 130603B, the X-ray emission became flattened^49^. The ratio between the macronova and X-ray radiation luminosities at the peak time of the macronova emission (that is, *R*_MN/X_) varies from burst to burst by up to a factor of 10. For sGRB 050709, hGRB 060614 and sGRB 130603B, the *R*_MN/X_ are ∼(1, 0.1, 0.4), respectively, which could shed some lights on the physical origin (see below).

A remarkable feature shown in Fig. 2 is the comparable peak luminosities of the different macronovae (that is, ∼10^41^ erg s^−1^). However, the macronovae associated with sGRB 050709 and hGRB 060614 were mainly identified in *I*/F814W-band, which are ‘bluer' than the F160W-band macronova component of sGRB 130603B. As in none of the cases we have a complete spectrum, it is not clear whether there was a real difference in the spectra.

## Discussion

The possible identification of three macronova candidates in a small sample containing just three or at most six events that are suitable for the search indicates that macronovae are common in sGRBs and hGRBs. Given the paucity of data for other events, macronovae could possibly arise in all shGRBs, although a macronova fraction as low as ∼0.18 can not be ruled out. A common feature of the macronova candidates is that the peak luminosity of macronovae is ∼10^40^–10^41^ erg s^−1^ in the optical to infrared bands with a timescale of 1 week. In the compact binary merger scenario of shGRBs, this can arise from dynamical ejecta with heavy *r*-process elements or lanthanide-free wind, or central engine activity. Here we discuss implications to each model.

The *I*-band light curve arising from dynamical ejecta with a mass of 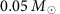 and an average velocity of 0.2*c* (see the black hole–neutron star merger model H4Q3a75 in ref. 17 and also ref. 55) is shown in Figs 1 and 2 as an example. Because of a fast expansion velocity and the large opacity of ≈10 cm^2^ g^−1^, the temperature is already low around the peak time and most of the photons are radiated in the near infrared *J*-, *H*- and *K*-bands. The luminosity in the *I*-band is smaller than that in the *H*-band by a factor of 3–10 at 1 week after merger. This model can reproduce the observed *I*-band data of sGRB 050709 and the hGRB 060614, and the *H*-band data of sGRB 130603B with 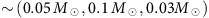, respectively. The massive ejecta with ≳0.05 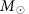 suggests that the progenitor of sGRB 050709 is a black hole–neutron star merger^34^,^35^,^36^,^37^. However, we should note that this estimate can changed by a factor of a few due to uncertainties in the opacity, nuclear heating and ejecta morphology. The upper limit in the *I*-band at 3 days of sGRB 061201 constrains the maximally allowed mass of dynamical ejecta as 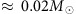 if the redshift of 0.111 is correct. The upper limits in the *I*-band of hGRB 060505 are both consistent with almost 0.05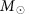 and can be even higher if absorption at the host galaxy was significant^52^. Interestingly, ref. 52 was the first to search for a macronova signature in the afterglow light curve of this burst.

The absence of lanthanides in a wind reduces the opacity. The resulting macronova has a brighter and bluer peak luminosity on a shorter timescale^13^,^14^,^47^,^48^,^56^. Figure 2 shows the *I*-band light curve arising from a lanthanide-free wind with a mass of 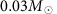 and an average velocity of 0.07*c*, where elements with atomic numbers of 31–54 are included (see the wind model in ref. 14 and also ref. 47). This model can reproduce the *I*-band data of GRB 050709 and 060614 at early times (*t*<5 days). However, the light curve at late times is faint compared with the data. Although increasing the wind mass raises the late *I*-band luminosity, such a model is too bright to be compatible with the early *I*-band data (*t*<5 days). The *I*-band upper limit at 3 days of GRB 061201 indicates the mass of a lanthanide-free wind of ≲0.01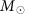. The upper limits on the afterglow of hGRB 060505 were taken much later after the bursts, and as such the implied limits on the wind ejecta are weak.

The central engine can also power a macronova. Here we focus on the X-ray-powered macronova model^57^, as this model is testable with the observed X-ray and optical data. In this model, X-ray photons emitted by the central engine are absorbed by the ejecta and re-emitted in the optical-infrared bands. It is noteworthy that *r*-process material with a mass of ≳10^−3^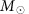 is required, to keep the ejecta optically thick to optical photons until 1 week after the merger. Although the spectrum and light curve of this emission are unclear, a relation of *L*_IR_≈0.1*L*_*X*_ (that is, *R*_MN/X_≈0.1) is expected in this scenario. As summarized in Table 1, *R*_MN/X_ varies among the events. In particular, for GRB 050709, it is difficult to explain the macronova luminosity with *R*_MN/X_≈1. However, the flare activity in X-ray at late times may provide enough energy to produce the *I*-band emission. Better data in both X-ray and optical infrared at late times are needed to further test the X-ray-powered macronova model.

The comprehensive examination of the near-infrared data of current nearby sGRBs and hGRBs yielded in total three or at most six events suitable for macronova searches. The successful identification of three candidates in such a limited sample demonstrates that macronovae arise in most, if not all, compact object merger events that produce GRBs. A comparison of the above three scenarios favours the by now ‘standard' dynamical ejecta that is enriched by *r*-process elements^11^,^35^,^58^. The massive *r*-process material ejecta inferred in each one of these events strongly suggest that compact object mergers are the significant or even prime sites of producing heavy *r*-process elements^3^,^38^,^39^,^40^,^41^,^42^,^43^,^44^.

These results have important implications on the future of macronovae and GW electromagnetic counterparts observations (for example, see refs 59, 60 for search strategies). Among the three macronova candidates, two were identified in *I*-band (there was also evidence for emission in *R*-band emission, too). Ground-based telescopes are much more sensitive in *I*-band than in *J*/*H*/*K*-bands. If the mergers powering sGRB 050709 and hGRB 060614 took place at luminosity distances of ∼200 Mpc (the horizon of advanced LIGO/Virgo network for double neutron star mergers) or ∼350 Mpc (the horizon of advanced LIGO/Virgo network for a neutron star merger with a ∼6 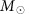 black hole), the corresponding peak *I*-band emission is expected to be as bright as ∼21–22th magnitude or ∼22–23th magnitude, respectively. Such events are marginally detectable by new and upcoming transient surveys such as the ESO VLT Survey Telescope (VST, https://www.eso.org/sci/facilities/paranal/telescopes/vst.html, see Abbott *et al.*^61^) and the Zwicky Transient Facility that is expected to have first light in 2017 (http://www.ptf.caltech.edu/ztf). The Large Synoptic Survey Telescope^62^ with a 9.6 deg^2^ field of view that can image about 10^4^ deg^2^ of the sky in three clear nights down to limiting magnitude of *i*∼23.5 (Vega system), in principle, could easily identify such signals.

## Methods

### Optical and infrared data reduction

**Figure 4:**
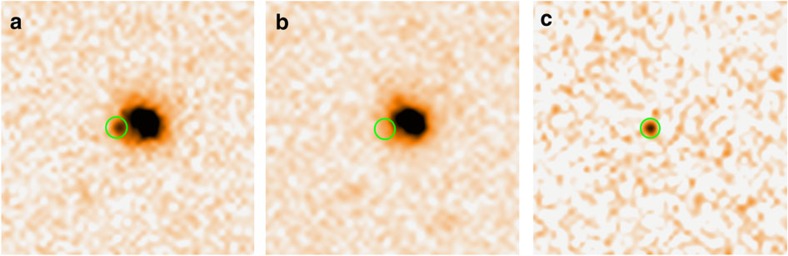
The VLT *I*-band images of the afterglow of GRB 050709. The data were taken on 12 July 2005 (**a**) and 30 July 2005 (**b**), and the signal resulted in the image substraction (**c**). The afterglow position has been circled and the afterglow emission is clearly visible on 12 July 2005. The images are magnified only for demonstration.

The VLT imaging data of GRB 050709 are publicly available in ESO Science Archive Facility (http://archive.eso.org). We reduce the raw data following the standard procedures in IRAF (http://iraf.noao.edu), including bias subtraction, flat fielding and image combination. Observations made with the same filter at different epochs are first aligned to the last epoch (reference frame), using the *imalign* tool in IRAF. The task *ficonv* in software package FITSH (http://fitsh.szofi.net) is used to convolve the reference to match the profile and brightness of objects in earlier frames. For each earlier frame, the reference frames is convolved to and subtracted. In this method, the object profile and zero point of the subtracted image are the same as the image that has been subtracted. Finally, the aperture photometry is applied to the residual images and find the instrumental magnitudes of the afterglow. Photometric errors are estimated from the photon noise and the sky variance to 1*σ* confidence level. The 3*σ* of the background RMS of the residual images is taken as the limiting magnitude. Eight- to ten-point-like objects in the field are used as reference stars for differential photometry. Finally, standard stars observed on 12 and 30 July 2005 were used for the absolute calibration. The results are presented in Table 2, consistent with that reported in the literature^31^. The main novel result is the detection of the *I*-band emission at *t*∼2.4 day after the trigger of the GRB (see Fig. 4). Our ‘new' detection is mainly benefited from the improvement on the change of the reference frame (that is, from 18 July FORS1 observation to 30 July FORS2 observation). The advantage is less ‘contamination' from emission of the source (about 25.2 Mag versus >27.2 Mag, according to the HST observation), the original and reference images are both from the same instrument (FORS2) on VLT. Hence, the signal-to-noise rate of the source is improved.

It is worth noting that in the direction of the burst, the Galactic extinction is expected to be just *E*(*B*−*V*)=0.01 mag^63^. The optical afterglow of GRB 050709 is superposed on the outskirts of the host galaxy and the extinction is probably very small (that is, ≤0.1 mag), too^27^. Therefore, in this work, we ignore the extinction corrections of the optical data. We have also analysed the Gemini-N *r*′-band data. In total, there are two sets of exposures (that is, 4 × 300 s on 16 July and 6 × 200 s on 28 July). However, the second sets of exposures have a high sky brightness (≈19.35 Vega Mag arcsec^−2^) that is not suitable for reference frame in the image subtraction. Therefore, we performed image subtraction between the high-quality Gemini-N (on 16 July) and VLT (on 30 July) observations and got an upper limit (see Table 2).

We downloaded the public HST archive data of GRB 050709 from the Mikulski Archive for Space Telescopes (http://archive.stsci.edu), including five observations with ACS in F814W-band. The reduced data provided by Mikulski Archive for Space Telescopes were used in our analysis. The last observation is taken as the reference and the other images of the same filter are subtracted, to directly measure fluxes of the afterglow from the residual images. Aperture photometry was carried out for the afterglow in the residual image. The ACS zeropoints were used for absolute calibration. If the signal of the afterglow is too faint to be a secure detection, an upper limit of 3*σ* background RMS is adopted. Our results are summarized in Table 2, nicely in agreement with Fox *et al.*^27^.

Danish 1.54 m telescope data are not publicly available and therefore we simply adopt the data reported in Hjorth *et al.*^30^. Table 2 is a complete list of the data points used in our analysis.

### Data availability

The HST, VLT and Gemini observation data analysed/used in this work are all publicly available (see Methods section).

## Additional information

**How to cite this article:** Jin, Z.-P. *et al.* The Macronova in GRB 050709 and the GRB to macronova connection. *Nat. Commun.* 7:12898 doi: 10.1038/ncomms12898 (2016).

## Supplementary Material

Supplementary Information Supplementary Table 1, Supplementary Discussion and Supplementary References.

## Figures and Tables

**Table 1:** Physical properties of GRBs/macronovae/afterglows with known redshifts.

	GRB 050709*	**GRB 060614**†	**GRB 130603B**‡
*E*_γ,iso_ (10^51^ erg)	0.069	2.5	2.1
*z*	0.16	0.125	0.356
Duration§ (s)	0.5 (+130)	5 (+97)	0.18
Classification	sGRB+extended X-rays	hGRB	sGRB
Identifying macronova	in *I*/F814W	in *I*/F814W	in F160W
Macronova peak luminosity	∼10^41^ erg s^−1^ (*I*)	∼10^41^ erg s^−1^ (*I*)	∼10^41^ erg s^−1^ (F160W)
*M*_ej_||	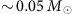	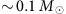	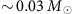
*R*_MN/X_¶	∼1	∼0.1	∼0.4

^*^Villasenor *et al.*^29^ and this work.

^†^Gehrels *et al.*^21^, Yang *et al.*^25^ and Jin *et al.*^26^.

^‡^Tanvir *et al.*^19^, Berger^20^ and Hotokezaka *et al.*^64^.

^§^The durations include that of the hard spike and the ‘extended emission' (in the bracket).

^||^The *M*_ej_ is estimated from the dynamical ejecta model and the value can change by a factor of a few due to uncertainties in the opacity, nuclear heating, and ejecta morphology.

^¶^*R*_MN/X_ denotes the ratio between the macronova ‘peak' luminosity and the simultaneous X-ray luminosity.

**Table 2:** The optical observations of GRB 050709.

**Time (days)**	**Exposure (seconds)**	**Filter**	Magnitude* **(Vega)**	**Flux (*****μ*****Jy)**	**Seeing (arcsec)**	**Sky brightness**†
2.46346	60 × 5‡	VLT/FORS2/V	24.45±0.15	0.59±0.08	0.76	21.79
4.36416	120 × 3	VLT/FORS1/V	>25.1	<0.31	0.89	21.55
10.48568	120 × 3	VLT/FORS1/V	>24.5	<0.55	0.73	19.49
20.16693	180 × 3	VLT/FORS2/V	—	—	0.66	21.46
2.47249	60 × 5	VLT/FORS2/R	23.80±0.08	0.90±0.07	0.68	21.19
6.64339	300 × 4§	Gemini-N/r′(R)	>25.4	<0.20	0.67	20.93
10.47943	120 × 3	VLT/FORS1/R	>24.6	<0.43	0.59	19.26
20.17874	180 × 15‡	VLT/FORS2/R	—	—	0.61	20.97
2.45513	100 × 6	VLT/FORS2/I	24.1±0.2	0.55±0.09	0.65	19.85
4.37179	100 × 3	VLT/FORS1/I	>23.6	<0.86	0.79	19.51
8.33429	120 × 10	VLT/FORS1/I	>24.8	<0.30	0.42	19.40
20.23152	180 × 3	VLT/FORS2/I	—	—	0.61	19.73
5.71410	6360	HST F814W	24.66±0.03||	0.330±0.009		
9.84385	6360	HST F814W	25.39±0.05||	0.169±0.008		
18.70269	6360	HST F814W	27.16±0.23||	0.033±0.008		
34.69556	6360	HST F814W	>27.4||	<0.026		
371.78780	7039	HST F814W	—	—		
1.4166	600 × 12	Danish/R	22.80±0.07¶	2.34±0.12		
2.3862	600 × 17	Danish/R	23.55±0.25¶	1.17±0.26		

^*^The magnitudes of the extracted optical transient, here magnitude errors are reported in 1*σ* and upper limits are 3*σ*.

^†^In units of Vega Mag arcsec^−2^.

^‡^Some images are not combined.

^§^The Gemini-N r′-band upper limit has been converted into *R*-band.

^||^Fox *et al.*^27^ reported the AB magnitudes, which are larger than the corresponding Vega magnitudes by 0.42 mag.

^¶^These data are taken from Hjorth *et al.*^30^.
