# Supplementary material for: The Macronova in GRB 050709 and the GRB-macronova connection
Source: Nat Commun. 2016 Sep 23;7:12898. doi: 10.1038/ncomms12898 (PMC5036160; doi:10.1038/ncomms12898)
Supplement: Supplementary Information — Supplementary Table 1, Supplementary Discussion and Supplementary References. [file ncomms12898-s1.pdf]

Supplementary Table 1. The nearby sGRBs and hGRBs with optical afterglow emission.

GRB	z	Ground-based I-band	HST nIR observations	Macronova search
050509B[a]	0.225		May 14, 18, 28 and Jun 1	Suitable (No)
050709[a,b]	0.161	VLT/Jul 12, 14, 18, 30	Jul 15, 19, 28 and later	Suitable (Yes)
050724[a,c]	0.257	VLT/Jul 25, 27, 30		Not Suitable
060502B[d]	0.287			Not Suitable
060505[e]	0.089	Gemini-S/May 14	May 19 and Jun 06	Suitable (No)
060614[f]	0.125	VLT/Jun 16, 17, 18 and later	Jun 28 and Jul 16	Suitable (Yes)
061201[a,g]	0.111	VLT/Dec 2, 3, 5, 18	Dec. 11	Suitable (No)
071227[a,h]	0.381			Not Suitable
080905A[a]	0.122			Not Suitable
130603B[a,i]	0.356	Gemini/Jun 4, 5	Jun 13 and Jul 03	Suitable (Yes)
140903A[a,j]	0.351	Gemini-N/Sep 5		Not Suitable
150101B[a,k]	0.134	VLT/Jan 4, 19, 20		Not Suitable

Note.

a. Fong et al. [1];

b. Fox et al. [2] and Covino et al. [3] ;

c. Malesani et al. [4];

d. Berger et al. [5];

e. Ofek et al. [6];

f. Della Valle et al. [7] and Gal-Yam et al. [8];

g. Stratta et al. [9];

h. D'Avanzo et al. [10];

i. Tanvir et al. [11];

j. Troja et al. [12];

k. Levan et al. [13].

Supplementary Discussion

The near-infrared observations of nearby sGRBs and hGRBs.

In the second part of Sec. II we have a sample of 12 nearby sGRBs and hGRBs. The near-infrared observations of these events are summarized in Supplementary Table 1. Note that for the afterglow emission of GRB 150101B, there is still no formally published paper, yet, and we collect the information from GCN Circulars as well as the websites for 8-10m class telescopes and HST. For other events we collect the data reported in the literature. Below we explain in some detail why we conclude that sGRB 060502B, sGRB 050724, sGRB 071227, sGRB 080905A, sGRB 140903A and sGRB 150101B are not suitable for macronova identification. We also describe the HST observations of the afterglow emission of sGRB 050509B since they have not been formally reported in the literature yet.

GRB 050509B: this burst had dense HST follow-up observations in F814W band on May 14, 18, 28 and June 1 2005 (http://archive.stsci.edu), each exposure lasted 6908 s. There is a very bright elliptical galaxy (~16 mag, z=0.225) near the *Swift* XRT error box, likely to be the host galaxy.

Without an identification of an optical counterpart, it is challenging to set a robust limit on the underlying macronova (see also [14] for similar conclusion on afterglow but based on the ground-based *R/V* observations). If the GRB is at the outskirt of the galaxy, the limit would be >27.4 mag and can be used to constrain the merger scenario. However if it is in the inner core of the galaxy, the limit would be >25.3 mag and the constraint on the macronova emission is weak.

sGRB 050724: this burst had rare ground-based I-band observations and no HST followup observation, the large amount of dust extinction (i.e., A_v=2 mag) [2, 4] in the direction of the burst is an important obstacle for macronova search, too.

sGRB 060502B: the redshift of this burst is suggested to be either unknown [5] or 0.287 [15]. Assuming a redshift of 0.287, the main obstacle for macronova hunting is the lack of dense and deep optical/nIR observations and there was just a *R*-band flux upper limit ≤0.7 μJy at ~16.8 hours reported [5]. Considering its relatively high redshift, a macronova signal, if intrinsically as luminous as that identified in sGRB 050709, can just give rise to *R*-band peak emission of ~26th mag, which is well below the single upper limit previously reported.

sGRB 071227: this event had a relatively high redshift (z=0.381) but had neither ground-based *I*-band observations nor HST follow-up observations [1].

sGRB 080905A: the redshift of z=0.122 is low that is suitable for macronova hunting. However, the latest two VLT/*R*-band follow-up observations were on Sept 7 and 23, respectively [1]. Such rare observations, either too early or too late, are not helpful for macronova search (This is in particular the case for the NS-NS merger scenarios for which the peak R-band macronova emission is expected to be ~26th mag for $M_{ej} \leq 0.01\ M_{\odot}$ and z=0.1 [16, 17]).

sGRB 140903A: this burst was at z=0.351, for which HST nIR observations are necessary to get the macronova signal. However, no HST exposure of the afterglow of GRB 140903A was performed, the available dataset is not deep enough to search for a macronova (also discussed in Troja et al. [12]).

sGRB 150101B: the redshift is z=0.134. If the associated kilonova emission is similar to that of GRB 130603B, a H-band peak magnitude is expected to be around H(AB)=22-23 [13], The IR observations with VLT/HAWK-I on January 16 however found no evidence for any source to a preliminary limiting magnitude of H(AB)>23.5 [18]. The macronova signal of GRB 130603B was detected at t~7 day (in the burster's rest frame), the non-detection in GRB 150101B in H-band may be due to the long delay of the exposure. The TNG had two J-band measurements on Jan 11, Jan 15 2015, respectively. The obtained upper limits, however, are not tight enough to exclude the presence of a macronova as bright as that of GRB 130603B [1]. The VLT I-band observations were performed either too early or too late for the macronova detection. The first visit of HST in F606W-band was on 11 Feb. 2015, which is about 40 days after the GRB trigger, too late to catch the macronova signal.

Supplementary References

[1] Fong, W. F. et al. A Decade of Short-duration Gamma-Ray Burst Broadband Afterglows: Energetics, Circumburst Densities, and Jet Opening Angles. *Astrophys. J.*, **815**, 102 (2015).

[2] Fox, D. B., et al. The afterglow of GRB 050709 and the nature of the short-hard γ-ray bursts. *Nature*, **437**, 845-850 (2005).

[3] Covino, S., et al. Optical emission from GRB 050709: a short/hard GRB in a star-forming galaxy. *Astron. Astrophys.*, **447**, L5-L8 (2006).

[4] Malesani, D., et al. Multicolor observations of the afterglow of the short/hard GRB050724. *Astron. Astrophys.*, **473**, 77-84 (2007).

[5] Berger, E. A Short Gamma-ray Burst "No-host" Problem? Investigating Large Progenitor Offsets for Short GRBs with Optical Afterglows. *Astrophys. J.*, **722**, 1946-1961 (2010).

[6] Ofek, E. O. et al. GRB 060505: A possible short-duration gamma-ray burst in a star-forming region at a redshift of 0.09. *Astrophys. J.*, **662**, 1129-1135 (2007).

[7] Della Valle, M. et al. An enigmatic long-lasting gamma-ray burst not accompanied by a bright supernova. *Nature*, **444**, 1050-1052 (2006).

[8] Gal-Yam, A. et al. A novel explosive process is required for the γ-ray burst GRB 060614. *Nature*, **444**, 1053-1055 (2006).

[9] Stratta, G., et al. A study of the prompt and afterglow emission of the short GRB 061201. *Astron. Astrophys.*, **474**, 827-835 (2007).

[10] D'Avanzo, P. et al. The optical afterglows and host galaxies of three short/hard gamma-ray bursts. *Astron. Astrophys.*, **498**, 711-721 (2009).

[11] Tanvir, N. R. et al. A `kilonova' associated with the short-duration gamma-ray burst GRB 130603B. *Nature*, **500**, 547-549 (2013).

[12] Troja, E. et al. An achromatic break in the afterglow of the short GRB 140903A: evidence for a narrow jet. *Astrophys. J.* in press, arXiv:1605.03573 (2016).

[13] Levan, A. J., et al. GRB 150101B/Swift J123205.1-105602: VLT observations and redshift. *GCN CIRCULAR*, 17281 (2015).

[14] Hjorth, J., et al. The optical afterglow of the short γ-ray burst GRB 050709. *Nature*, **437**, 859-861 (2005).

[15] Berger, E. Short-Duration Gamma-Ray Bursts. *Ann. Rev. Astron. Astrophys.*, **52**, 43-105 (2014).

[16] Kasen, D., Badnell, N. R. and Barnes, J., Opacities and spectra of the r-process ejecta from neutron star mergers. *Astrophys. J.*, **774**, 25 (2013).

[17] Tanaka, M., and Hotokezaka, K. Radiative Transfer Simulations of Neutron Star Merger Ejecta. *Astrophys. J.*, **775**, 113 (2013).

[18] van der Horst, A. J., et al. GRB 150101B/Swift J123205.1-105602: continued WSRT radio and VLT optical observations. *GCN CIRCULAR*, 17309 (2015).
